# Role of ncRNAs in the Pathogenesis of Sjögren’s Syndrome

**DOI:** 10.3390/biomedicines12071540

**Published:** 2024-07-11

**Authors:** Amal Al-Haidose, Sondoss Hassan, Mahmoud Elhassan, Eiman Ahmed, Abdulla Al-Riashi, Yazeed M. Alharbi, Monther Ghunaim, Talal Alhejaili, Atiyeh M. Abdallah

**Affiliations:** 1 Department of Biomedical Sciences, College of Health Sciences, QU Health, Qatar University, Doha 2713, Qatar; amalah@qu.edu.qa (A.A.-H.); sh1608327@student.qu.edu.qa (S.H.); mm1805337@student.qu.edu.qa (M.E.); ea1208168@student.qu.edu.qa (E.A.); aa2300103@student.qu.edu.qa (A.A.-R.); 2 Department of Internal Medicine, Collage of Medicine, Taibah University, Madinah 42353, Saudi Arabia; tu4303138@taibahu.edu.sa (Y.M.A.); mghunaim@taibahu.edu.sa (M.G.); 3 Department of Gastroenterology, King Salman Medical City, Madinah 42319, Saudi Arabia; tlalhejaili@moh.gov.sa

**Keywords:** RNA expression, non-coding RNAs, Sjögren’s syndrome, autoimmune disease

## Abstract

Sjögren’s syndrome is a multisystemic autoimmune disease that mainly affects the exocrine glands, causing dryness of the eyes and the mouth as the principal symptoms. Non-coding RNAs (ncRNAs), once regarded as genomic “junk”, are now appreciated as important molecular regulators of gene expression, not least in Sjögren’s syndrome and other autoimmune diseases. Here we review research into the causative roles of microRNAs (miRNAs), long non-coding RNAs (lncRNAs), and circular RNAs (circRNAs) on immunological responses, inflammation, and salivary gland epithelial cell function in Sjögren’s syndrome patients. These ncRNAs represent promising new therapeutic targets for treating the disease and possibly as biomarkers for early diagnosis.

## 1. Introduction

### 1.1. Sjögren’s Syndrome

Sjögren’s syndrome (SS) is a complex and relatively common autoimmune disease that affects millions of people worldwide [1]. It is characterized by immune-mediated dysfunction of exocrine glands [2], especially the lacrimal and salivary glands, which gives rise to xerostomia (dry mouth) and xerophthalmia (dry eye), respectively, although extra-exocrine symptoms are also common [3]. SS has classically been divided into primary (pSS) and secondary (sSS) types [4]. pSS is an independent autoimmune disease that largely affects the exocrine glands and sometimes other organ systems but not in the presence of other autoimmune diseases [5]. In sSS, systemic lupus erythematosus (SLE) and rheumatoid arthritis (RA) commonly coexist [6].

SS is far more common in females than in males (female-to-male ratio of almost 9:1) [3], prompting investigations into the possible underlying hormonal, genetic, or immunological mechanisms underpinning its pathogenesis. Furthermore, although SS can manifest at any age, it is more common in people between 40 and 60 years [1]. There is also notable geographic variability in the incidence of SS, suggesting a multifactorial pathoetiology [7,8].

**Figure 1:**
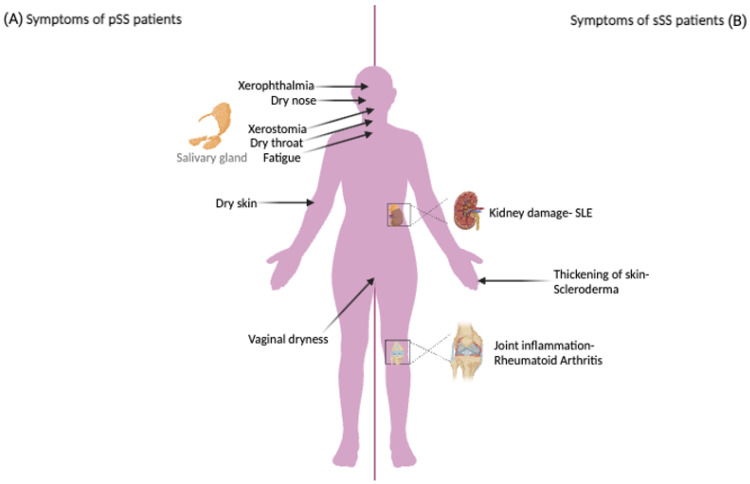
The main symptoms characterizing pSS and sSS. The main symptoms in pSS are xerophthalmia and xerostomia (**A**), while the main symptoms in sSS are kidney damage (associated with SLE) and joint inflammation (associated with RA) (**B**). Figure created with BioRender.com.

Dry eyes, or xerophthalmia, is common and clinically challenging [7]. Reduced tear production causes eye dryness and irritation, which can cause pain, blurred vision, and an increased risk of eye infections [8,9]. Simultaneously, reduced salivation results in xerostomia, or dryness of the mouth and throat [10]. Early identification and treatment are important, as xerostomia raises the risk of dental complications and makes swallowing, speaking, and tasting food harder. SS is, however, multisystemic, giving rise to numerous clinical symptoms including joint pain, severe exhaustion, skin rashes, and sometimes organ disease [7]. Effective patient care therefore requires complete awareness of the multifaceted symptomatology of SS [11] and how this impacts the quality of life and well-being of those suffering from the condition [7]. Histopathologically, affected glands tend to contain lymphocytic infiltrates [11] that contribute to gland damage and dysfunction [12] (Figure 1).

### 1.2. Non-Coding RNAs

Non-coding RNAs (ncRNAs), which include circular RNAs (circRNAs), long non-coding RNAs (lncRNAs), and microRNAs (miRNAs) [13], are now recognized as essential participants in autoimmune diseases [14]. Although the focus of this review is SS, it is important to understand how ncRNAs affect autoimmunity more broadly [15]. ncRNAs describe a broad class of RNA molecules that actively control gene expression but do not encode proteins. miRNAs are short RNA sequences (19–25 nucleotides in length) that regulate target gene expression, and their dysregulation has been linked to several autoimmune diseases [14], so they may therefore be potential diagnostic and therapeutic targets. LncRNAs are similarly linked to various autoimmune disorders, playing regulatory roles in tissue damage, inflammation, and immunological responses [14]. The discovery of lncRNAs unique to a certain diseases might similarly provide opportunity for specific diagnostics and personalized treatment strategies [13]. By virtue of their covalently closed loop structure, circRNAs are especially stable ncRNAs [16], making them attractive diagnostic candidates.

Given their established roles in other autoimmune disorders, we reviewed the roles played by ncRNAs in SS, aiming to present a thorough overview of how these ncRNA molecules are involved in the etiopathogenesis of the disease and hence their potential as diagnostic and therapeutic targets (Table 1). In this review, we focused on data from studies involving SS patients rather than from animal models.

## 2. miRNAs in Sjögren’s Syndrome

### 2.1. miRNA Expression in the Exocrine Glands

SS is characterized by severe exocrine gland dysfunction and dysfunction [38] associated with alterations in calcium signaling. Jang et al. sequenced primary human salivary gland epithelial cell cultures (phSG) grown in keratinocyte growth medium (KGM) supplemented with low or high calcium, which resulted in the upregulation of miR-1248 in the high-calcium culture conditions. Furthermore, increased miR-1248 expression was associated with IFN activation through binding with argonaute (AGO)-2 and retinoic acid-inducible gene I (RIG-I), also known as DExD/H-box helicase (DDX)-58, in SS patients compared to normal patients [17]. Other studies have explored the role of miRNAs in regulating the salivary glands in SS patients. For example, Carvajal et al. quantified the expression of Homo sapiens (hsa)-miR-424–5p and hsa-miR-513c-3p and identified their targets in labial salivary gland (LSG) biopsies from pSS patients. hsa-miR-424–5p expression was downregulated while hsa-miR-513c-3p expression was upregulated in pSS patients compared with controls. The activating transcription factor (ATF)-6αlpha and protein sel-1 homolog 1 (SEL1L) were identified as hsa-miR-424–5p targets, which were upregulated in tissue biopsies, and the overexpression of hsa-miR-424–5p resulted in the downregulation of ATF6α and SEL1L. By contrast, XBP-1s and GRP78 were identified as hsa-miR-513c-3p targets, which were downregulated in tissue biopsies, while silencing hsa-miR-513c-3p increased the spliced form of X-box binding protein 1 (XBP-1s) and 78 kDa glucose-regulated protein (GRP78) expression [18]. Wang et al. investigated the role of miRNAs in SS by applying microarray profiling to labial salivary glands (LSGs) from patients with SS, finding that miR-181a and miR-16 were downregulated in SS patients compared with controls. Moreover, they reported a correlation between these two miRNAs and Ro/SSA and La/SSB, intracellular ribonucleoproteins used mainly as therapeutic targets for autoimmune humoral treatments, in SS patients [19]. However, the authors did not investigate the exact mechanism by which these miRNAs might contribute to SS pathogenesis via Ro/SSA and La/SSB.

miRNAs can modulate pathological processes by targeting different cellular pathways, but the exact biochemical pathways involved in pSS are still unknown. One pathway implicated in SS is the mucin-type O-glycan biosynthesis pathway. Gallo et al. assessed miRNA and gene expression in SS patients with high and low salivary flow, with unaffected individuals used as a control. Minor salivary gland biopsies (MSGBs) were collected from five participants. Using quantitative reverse transcriptase polymerase chain reaction (qRT-PCR) assays, 126 miRNAs were significantly dysregulated compared with the controls, where four were upregulated (hsa-miR-106a, hsa-miR-146b, hsa-miR-18b, hsa-miR-20a) and two were downregulated (hsa-miR-372, hsa-miR-635). The expression of these miRNAs was inversely proportional to salivary flow rates. In addition, there was an overexpression of miR-30, miR-17/92, miR-200, and miR-let-7 members in pSS patients. Pathway analysis revealed that these dysregulated miRNAs participate in the mucin-type O-glycan biosynthesis pathway. An in silico approach was used to identify affected genes and, using qRT-PCR, a decrease in genes coding for glycosyltransferases and glycosidases was detected. One important gene downregulated due to miR-let-7 overexpression was polypeptide N-Acetylglucosaminyltransferase, (*GALNT*)*-1*, which encodes the mucin (MUC)-7 salivary protein and is responsible for oral lubrication [39]. Other downregulated genes included *GALNT1*, *GALNT2*, *GALNT4*, beta-1,4-galactosyltransferase 5 (*B4GALT5*), and others. Glycosylated mucin is an important salivary constituent, so dysregulation of the mucin-type O-glycan biosynthesis pathway might contribute to the symptoms experienced by patients [20].

The mucin-type O-glycan biosynthesis pathway is highly conserved across species. hsa-miR-181d-5 is another miRNA reported to affect downstream genes in the mucin-type O-glycan biosynthesis pathway, such as *GALNT4*, *GALNT10*, Complement C1q A Chain (*C1GA*), and other polypeptide N-acetylgalactosaminyltransferase (GalNAc-T) family proteins. This miRNA also regulates pathways involved in endoplasmic reticulum (ER) stress and TNF-α and other pro- and anti-inflammatory pathways [21]. Castro et al. explored the relationship between hsa-miR-181d-5 and TNF-α in LSG samples collected from control and pSS patients. Using next-generation sequencing technologies and qRT-PCR, they identified and validated a list of miRNAs together with their respective pathway candidates implicated in glandular inflammation. They focused on the most significantly differentially expressed miRNA, hsa-miR-181d-5p and, using Spearman’s analysis, discovered an inverse correlation between hsa-miR-181d-5p and TNF-α expression. The increase in TNF-α may explain the dysregulated glandular function and morphology observed in pSS patients, as TNF-α is implicated in altered cell polarity and acinar structure in the salivary glands [40]. Given this association, hsa-miR-181d-5p may be a novel therapeutic target in SS [21].

Interferons (IFN) are important regulators of immune responses, including in autoimmune disease. Type 1 IFNs affect the expression of specific inflammatory genes to produce so-called type 1 IFN signatures (MX Dynamin Like GTPase 1 (*MX1*), interferon induced protein with tetratricopeptide repeats (*IFIT*)*-1*, *IFI44*, and *IFI44L*) in pSS patients. Jara et al. investigated the effect of the type 1 IFN pathway on hsa-miR-145-5p expression and the influence of hsa-miR-145-5p on its target mucin 1 (*MUC1*) and toll-like receptor 4 (*TLR4*) genes, as well as other type 1 IFN signature genes. *MUC1* and *TLR4* overexpression contributes to the pathogenesis of pSS [22], where the overexpression of *MUC1* can lead to ER stress in LSG cells and increase proinflammatory cytokines and inflammation [41], contributing to disease pathogenesis. Similarly, overexpression of *TLR4* causes ER stress, which triggers an increase in pro-inflammatory cytokine localization [42]. In LSG biopsies collected and analyzed from pSS patients and unaffected controls, type 1 IFN genes, *MUC1* and *TLR4* were upregulated in pSS patients compared with controls [22]. Conversely, the downregulation of hsa-miR-145-5p has been observed in pSS patients compared with unaffected controls [43], suggesting a negative association between type 1 IFN signature genes *MUC1* and *TLR4* with hsa-miR-145-5p. In confirmatory in vitro studies using cultured HSG cells, HSG cells treated with 10 ng/mL IFN-α and IFN-β exhibited a significant decrease in hsa-miR-145-5p expression, which was associated with high concentrations of *MUC1* and *TLR4*. To confirm whether hsa-miR-145-5p regulates *MUC1* and *TLR4* expression, HSG cells were transfected with an miRNA mimic or inhibitor, which confirmed a decrease in *MUC1* and *TLR4* in the former and an increase in the latter [22]. In conclusion, hsa-miR-145-5p may be a target to regulate pro-inflammatory gene expression and inflammation in glands affected in pSS.

### 2.2. miRNAs in Peripheral Blood

The infiltration of lymphocytes, including T cells and T cell subsets, into the salivary and lacrimal glands is a major hallmark of SS [44]. Indeed, T cell infiltration in SS patients is correlated with disease activity and higher mortality [45]. Several studies have demonstrated a role for miRNAs in the lymphocytic infiltration seen in SS. For example, Gao et al. assessed the miRNA expression associated with lymphocyte infiltrates in pSS, with differential gene expression analysis revealing a downregulation of hsa-miR-3202 in pSS patients compared with controls. To investigate the role of hsa-miR-3202 in T cell infiltrates and infiltration, the authors transfected Jurkat T cells with miR-3202 mimics or inhibitors, which revealed a reduction in lymphocyte infiltration with miR-3202 mimics and the opposite with inhibitors. The authors identified matrix metalloproteinase-2 (*MMP2*) as a potential miR-3202 target, which is known to play a role in T cell invasion. To study the effect of miR-3202 on MMP expression, Jurkat T cells were transfected with miR-3202 mimics or its expression was inhibited, which confirmed a reduction in *MMP2* with miR-3202 mimics and an increase with inhibition [23].

Another study identified miRNAs regulated in IFN-mediated immune responses in systemic autoimmune diseases, where SS. Johansson et al. measured miR-31-5p and miR-150-5p levels in different T cell populations (CD3^+^, CD14^+^, CD15^+^, and CD19^+^) after intramuscular injection of IFN-β therapy. In T cells isolated from these patients, the miR-31-5p levels decreased while the miR-150-5p levels were unaltered when compared with healthy controls, indicating that miR-31-5p is reduced in T cells during acute and chronic type I IFN reactions while miR-150-5p is only reduced in acute type I IFN reactions [24].

There have also been studies of the role of T-helper (Th)17 in SS. Wang et al. found that miR-146a-5p was upregulated in Th17 cells and that it positively regulated Th17 differentiation in pSS patients compared with healthy controls. To study the mechanism by which miR-146a-5p enhances Th17 differentiation, Wang et al. mimicked and inhibited miR-146a-5p in peripheral blood mononuclear cells (PBMCs) derived from pSS patients. Inhibiting miR-146a-5p decreased the percentage of Th17 cells together with expression of the Th17-associated cytokines IL-17 and IL-21 and the IL-23 receptor (IL-23R) and STAT3. On the other hand, transfecting PBMCs with miR-146a-5p mimics increased the proportion of Th17 cells and their related cytokines. Furthermore, miR-146a-5p negatively regulated its target A disintegrin and metalloprotease (*ADAM)-17* [25], which itself inhibited the IL23/STAT3 pathway through IL-23R [46]. Wang et al. further investigated if this also applies in SS patients. As expected, miR-146a-5p inhibition increased ADAM17 levels [25]. Pauley et al. investigated the role of another miR-146 family member, miR-146a, in SS pathogenesis in patients and SS-prone mouse models. A functional assay using THP-1 cells showed an upregulation of miR-146a in PBMCs from SS patients compared with controls as well as in PBMCs and salivary glands from SS-prone mice. miR-146a also increased phagocytosis, and inhibiting miR-146a resulted in a significant reduction in phagocytosis compared with the controls. Furthermore, miR-146a suppressed the production of inflammatory cytokines, including TNF-α, IL-6, IL-1β, monocyte chemoattractant protein-1alpha (MIP-1α), and interferon gamma inducible protein-10 (IP-10). These findings suggest a role for miR-146a in innate immunity [26]. A follow-up study carried out by Gauna et al. aimed to identify miR-146a targets, and the results showed that miR-146a inhibited its potential target CD80 in the salivary gland. Decreased CD80 levels in the salivary gland can directly prevent effector T cell and Treg activation [27]. Similarly, Talotta et al. detected higher expression of miR-146a in salivary glands from SS patients compared with controls [47]. Other studies have reported that miR-146a affects immune responses in SLE by upregulating type 1 IFN and toll-like receptors (TLR) and promoting innate immune responses [48].

Since IL-17 plays a role in the pathogenesis of SS, Wang et al. investigated the effect and mechanism by which miR-let-7d-3p might regulate IL-17 expression in CD4^+^ lymphocytes isolated from the PBMC fraction. IL-17 was significantly higher in pSS patients than the controls, as confirmed by an enzyme-linked immunosorbent assay (ELISA). Gene and protein expression of IL-17 were inversely proportional to miR-let-7d-3p expression. Furthermore, consistent with the AKT1/mTOR pathway regulating IL-17 in various autoimmune diseases, the miR-let-7d-3p knockout increased IL-17 levels via the AKT1/mTOR pathway, and co-overexpressing AKT1 with miR-let-7d-3p recovered IL-17 expression [28].

Conventional dendritic cells (cDC) play an important role in the immune system by interacting with T cells [6]. Lopes et al. examined differential miRNA expression in circulating type-2 cDC2s obtained from pSS patients and healthy controls. miR-130a and miR-708 were downregulated in pSS patients in both training and validation cohorts. Mitogen- and stress-activated protein kinase (MSK1), which regulates the production of proinflammatory cytokines, was identified as a novel miR-130a target and was upregulated in cDC2s from pSS patients and in TNF-α- and IL-12-producing cells. miR-130a overexpression reduced MSK1 expression in cDC2s, and MSK1 inhibition decreased activation of cDC2s and IL-6, TNF-α, and IL-12 production [29].

Another hallmark of SS is infiltration of tissues with B cells, and many changes in their phenotype have been observed [49]. Wang-Renault et al. studied the expression profiles of B cells isolated from the salivary glands of pSS patients using qRT-PCR. They found that hsa-mir-30b-5p was downregulated in B cells in pSS patients compared with control samples, and hsa-mir-30b-5p expression in B cells was inversely correlated with B cell-activating factor (BAFF), which harbors an hsa-miR-30b-5p binding site in the 3′UTR. To confirm this, they went on to transfect THP-1 (a human leukemia monocytic cell line) cells with hsa-miR-30b-5p inhibitor, which increased BAFF as expected [30]. Upon binding to its receptor, BAFF promotes C cell proliferation, differentiation, and survival [50]. Increased BAFF expression was also associated with autoimmune disease development in mice [51].

**Figure 2:**
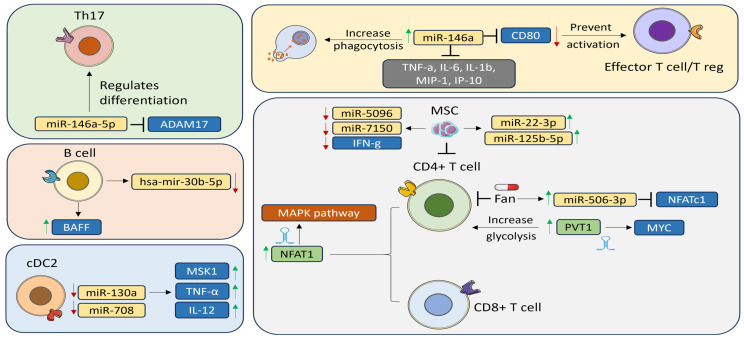
Many miRNAs play a critical role in pSS (primary Sjögren’s Syndrome) pathogenesis by regulating immune cells. MiR-146a-5p positively regulates Th17 differentiation in pSS patients and inhibits ADAM17 (A disintegrin and metalloprotease 17). These cells show a downregulation of mir-30b-5p and upregulation of B cell activating factor (BAFF), which has a binding site for miR-30b-5p in the 3′UTR. This shows the negative correlation between BAFF and miR-30b-5p. Another infiltrating immune cell is the type-2 conventional dendritic cells (cDC2s). MiR-130a and miR-708 are downregulated in cDC2s extracted from pSS patients. MiR-130a targets MSK1 (Mitogen- and stress-activated protein kinase), a protein that controls the production of proinflammatory cytokines. MSK1 is upregulated in pSS patients in addition to TNF-α and IL-12. MiR-146a is upregulated in pSS patients and plays a role in disease pathogenesis through the inhibition of its target CD80 in the salivary gland. Consequently, the decreased levels of CD80 can directly inhibit the activation of effector T cells and Tregs activation. Moreover, miR-146a represses the production of inflammatory cytokines including TNF-a, IL-6, IL-1b MIP-1a, and IP-10. Moreover, it reduces the levels of miR-5096, miR-7150, and IFN-g, while elevating the levels of miR-22-3p and miR-125b-5p. Another treatment that inhibits CD4^+^ T cells proliferation is fangchinoline (Fan) via upregulating miR-506-3p, which suppress NFATc1 (Nuclear factor of activated T cells 1) gene. lncRNAs also play a role in SS pathogenesis. PVT1 (plasmacytoma variant translocation 1) expression is high in pSS patients and increases glycolysis of activated CD4^+^ T cells and regulates the expression of MYC (myelocytomatosis oncogene). Another lncRNA is nuclear paraspeckle assembly transcript 1 (NEAT1), which has higher expression in CD4^+^ T cells and CD8 T cells in pSS patients.

Inflammatory chemokines and cytokines, such as Rantes and chemokine interferon-γ inducible protein 10 kDa (CXCL10), are significant contributors to the pathogenesis of pSS, including through miRNA-mediated mechanisms. In primary human conjunctival epithelial cells (PECs) from pSS patients, miR-744-5p was significantly upregulated compared with control samples. This miR-744-5p upregulation was associated with the downregulation of Pellino3 (*PELI3*), a negative regulator of inflammation. To validate the miR-744-5p/PELI3 relationship, pectoral muscles (PECs) isolated from pSS patients were treated with miR-744-5p antagomir, which resulted in downregulation of miR-744-5p and increased expression of PELI3 [31]. In a separate experiment, PECs collected from controls were transfected with miR-744-5p mimic, which significantly reduced expression of PELI3. An increase in PELI3 reduced IFN-dependent chemokines Rantes and CXCL10, which significantly contributed to disease progression in mouse models [52]. In addition, miR-744-5p has been reported to be involved in SLE, where it plays a role in ocular surface inflammation via PELI3 expression [31] (Figure 2).

### 2.3. Targeting miRNAs

miRNAs can be targeted therapeutically. Shao et al. investigated the biological mechanism by which fangchinoline (Fan), a natural extract from *Stephania tetrandra* S. Moore used in traditional Chinese medicine for rheumatic diseases, inhibits the CD4^+^ T cells in SS mice. Fan reduced CD4^+^ T cell infiltration and mitigated mouth dryness in SS mice. In addition, Fan inhibited CD4^+^ primary T cell proliferation in vitro. Moreover, NFATc1 expression was suppressed by miR-506-3p upregulation [53]. Mesenchymal stem cells (MSCs) are thought to suppress CD4^+^ T cells in pSS patients and mouse models, but the underlying mechanisms are uncertain [54]. Gong et al. performed miRNA analysis of CD4^+^ T cells treated with MSCs and found that MSCs inhibited CD4^+^ T cells and reduced IFN-γ levels. In addition, elevated miR-5096 and miR-7150 and reduced miR-22-3p and miR-125b-5p expressed by activated CD4^+^ T cells from SS patients were reversed upon MSC treatment [55]. In atherosclerosis, miR-22-3p decreased the inflammatory response by promoting the M2 anti-inflammatory macrophage phenotype and inhibited activation of NLRP3 through JAK1 [56].

## 3. LncRNAs in Sjögren’s Syndrome

There is accumulating evidence that lncRNA dysregulation may induce or mediate autoimmune diseases due to their roles in immune cell formation, activation, and function (Table 2) [32]. Fu et al. analyzed differentially expressed lncRNAs in SS lesions and found that PVT1 (plasmacytoma variant translocation 1) interacts with myelocytomatosis oncogene (Myc) to increase glycolysis upon CD4^+^ T cell activation. The lncRNA *PVT1*, a well-known non-Hodgkin B cell lymphoma oncogene, interacts with Myc, the expression of which is elevated in SS lesions. *PVT1*-mediated downregulation of Myc inhibits activation-induced glycolysis and glutaminolysis, which disrupts glucose and glutamine consumption and delays energy synthesis needed for proliferation. *PVT1* has also been implicated in dysregulated cells in rheumatoid arthritis patients, including FLSs (fibroblast-like synoviocytes). Downregulation of *PVT1* reduces proliferation and IL-1β release while inducing apoptosis of rheumatoid arthritis fibroblast-like synoviocytes (RA-FLS) by mediating the miR-543/SCUBE2 axis [57].

The lncRNA nuclear paraspeckle assembly transcript 1 (NEAT1), associated with immunity, regulates cytokine production, immune responses, and inflammasome stimulation. NEAT1 may be a novel lncRNA immunoregulatory factor that stimulates chemokine and interleukin secretion to affect monocyte–macrophage functions and T cell differentiation. NEAT1 plays a critical role in immune response control. In pSS patients, NEAT1 expression was predominantly increased in peripheral T cells, including CD4^+^ and CD8^+^ T cells, and this expression was positively correlated with disease progression. NEAT1 expression is positively correlated with clinical symptoms in SLE patients and was noticeably increased, mainly in monocytes. Lipopolysaccharide (LPS) can induce NEAT1 expression by activating p38, and the expression of NEAT1-induced factors can be affected by NEAT1 through the TLR4–MAPK pathway [33]. NEAT1 also enhances CD4^+^ T cell differentiation into Th17 cells in RA by increasing STAT3 expression, a key Th17 differentiation factor [58]. In LSGs, the lncRNAs ENST00000420219.1, ENST00000455309.1, n336161, NR 002712, ENST00000546086.1, Lnc-UTS2D-1:1, n340599, and TCONS l2 00014794 were elevated in pSS to possibly impact chemokine signaling pathways, TNF signaling, nuclear factor-kappa B (NF-κb) signaling, and natural killer cell-mediated cytotoxicity, all of which are pathological mechanisms in pSS. ENST00000455309.1 showed the strongest correlation with disease course, erythrocyte sedimentation rate (ESR), rheumatoid factor (RF), and IgA expression levels in pSS [37].

Many lncRNAs were identified as differentially expressed in PBMCs from pSS patients. BST2 Interferon Stimulated Positive Regulator (*BISPR*), Cytoskeleton Regulator RNA (*CYTOR*), *LINC00426*, Negative Regulator of Interferon Response (*NRIR*), and tensin homology pseudogene 1 (*TPTEP1*)*-202* were expressed and highly associated with pSS disease activity. Both *BISPR* and *NRIR* interacted with co-localized and co-expressed mRNAs implicated in NF-κB, JAK-STAT, and other signaling pathways that control cell movement. High mobility group box 1 (*HMGB1*), which is found downstream of LINC00426, is a DNA-binding nuclear protein linked to inflammatory diseases that might activate innate immune cells (macrophages/monocytes) via TLR-2/4 contact to cause the release of proinflammatory cytokines such as IL-8 via the NF-κB or MAPK signaling pathways [59]. These immune cells, inflammatory cytokines, and signaling pathways have been linked to the pathophysiology of pSS [34]. Dolcino et al. discovered three lncRNAs, CTD-2020K17.1, LINC00511, and LINC00657, with gene targets involved in the pathogenesis of SS through T cell differentiation, type 1 interferon and inflammation responses, and B cell physiology and malignancy. LINC00657 regulated the genes associated with cell adhesion, epithelial cell polarization, and apoptosis, consequently regulating the genes associated with inflammation, T cell activation and development, and B cell activity. LINC00511 was reported to regulate apoptosis, with anti-apoptotic BRI1-associated kinase 1 (*BAK1*), which is highly expressed in B cell lymphoma, regulated by CTD-2020K17.1 [60].

**Table 2:** Common long non-coding RNAs involved in autoimmune diseases.

LncRNA	Immune Response	Actions	Associated Diseases	Ref.
*PVT1*	Upregulated	Interacted with Myc to increase glycolysis upon CD4^+^ T cell activation	SS	[32]
	Downregulated	Bound to miR-543, which negatively regulated SCUBE2 expression	RA	
*NEAT1*	Upregulated	Stimulated chemokine and interleukin secretion to affect monocyte–macrophage functions and T cell differentiation	pSS	[33]
			SLE	
		Improved CD4^+^ T cell differentiation into Th17 cells by increasing STAT3 expression	RA	
*NRIR* and *BISPR*	Upregulated	Strongly correlated with JAK–STAT signaling, impacting as a negative regulator of IFN responses and resulting in an increase in the type 1 IFN-stimulated transcription	pSS	[34]
*LINC00426*	Downregulated	Had an impact on HMGB1 located downstream that could activate the innate immune cells (macrophages/monocytes) through interaction with TLR-2/4 and trigger the release of cytokines like IL-8 through the NF-κB or MAPK signaling pathways	pSS	[34]
ENST00000455309.1	Upregulated	Correlated with the β2 microglobulin expression. Involved in chemokine signaling, TNF signaling, NF-κB signaling, and natural killer cell-mediated cytotoxicity	pSS	
*LINC00657*	Upregulated	Regulated large number of genes involved in cell adhesion, epithelial cell polarization, and apoptosis to regulate genes involved in T cell development and activation including the genes related to B cell activity	pSS	[60]
*LINC00511*	Upregulated	Regulated a large number of transcripts involved in apoptosis	pSS	[60]
*CTD-2020K17.1*	Upregulated	Regulated *BAK1*, which is frequently overexpressed in B cell lymphomas	pSS	[60]

Abbreviations: Ref: References, SS: Sjögren’s syndrome, pSS: Primary Sjögren’s syndrome, RA: Rheumatoid arthritis, SLE: Systemic Lupus Erythematosus, NEAT1: Nuclear enriched abundant transcript 1, SCUBE2: Signal peptide, CUB domain and EGF like domain containing 2, SLE: Systemic lupus erythematosus, NRIR: Negative regulator of interferon response, BISPR: BST2 interferon stimulated positive regulator, BAK1: BRI1-associated kinase 1.

## 4. Circular RNAs in Sjogren’s Syndrome

**Figure 3:**
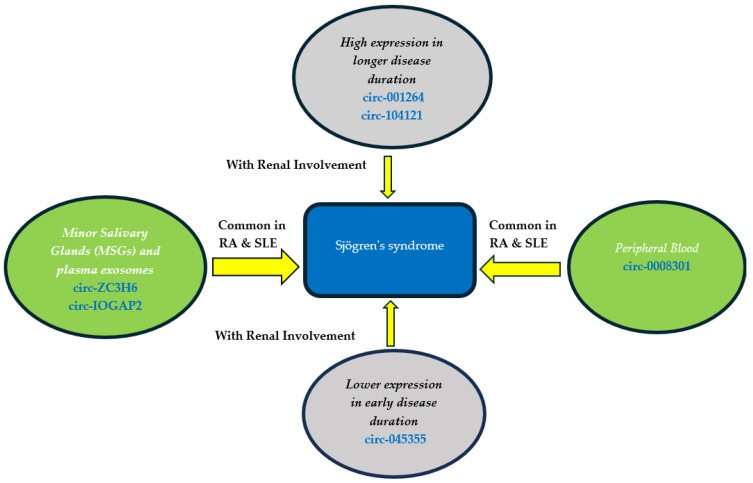
Relationship between circRNAs and pSS.

Circular RNAs (CircRNAs) are important gene regulators closely related to the development, progression, and severity of autoimmune diseases [36]. CircRNAs may act as miRNA sponges through their miRNA binding sites, thereby altering miRNA function. Li et al. used RNA-seq and qRT-PCR to quantify circRNAs in the minor salivary glands and plasma exosomes from pSS patients and non-pSS individuals. Only circ-ZC3H6 and circ-IQGAP2 were upregulated in exosomes, and their expression correlated significantly with clinical features, serum IgG levels, and MSG focus scores. The study therefore explored the possible utility of circ-IQGAP2 and circ-ZC3H6 as noninvasive biomarkers for the diagnosis of pSS, and expression of these two circRNAs in plasma exosomes were significantly higher in SS and in other autoimmune diseases, such as RA and SLE, perhaps due to a common pathoetiology [35]. pSS patients with early disease showed significantly higher expression of hsa_circRNA_001264 and hsa_circRNA_104121 compared with patients with longer disease duration and those with renal involvement and arthritis. In contrast, hsa_circRNA_045355 expression was lower in early pSS patients and those with renal involvement. Expression of hsa_circRNA_001264, hsa_circRNA_104121, and hsa_circRNA_045355 was strongly related to some clinical features, laboratory parameters, and disease activity indices in pSS patients [61]. Furthermore, circulating hsa_circ_0008301 was upregulated in early pSS patients. Toll-interacting protein (TOLLIP), the parent gene of hsa_circ_0008301, is a ubiquitous intracellular adapter protein that strongly participates in intracellular signaling pathways related to human diseases. Furthermore, TOLLIP plays a key role in mediating intracellular inflammatory responses, promoting autophagy, and facilitating intracellular vacuolar transport. TLR7 (Toll-like receptor 7) contributes to thrombocytopenia associated with pSS as manifested by the relatively higher expression level of hsa_circ_0008301 in patients with thrombocytopenia (Figure 3).

## 5. Conclusions

To the best of our knowledge, this is the first review discussing the role of miRNAs, lncRNAs, and circRNAs in SS. This in-depth look at SS, a complicated autoimmune disorder, shows how ncRNAs may play a key role in its development through regulation of gene expression, inflammation, and immune responses, which, in turn, affect the salivary gland phenotype. The investigation of ncRNAs in SS provides new insights into the mechanisms underlying this complicated autoimmune disease. This review not only broadens our knowledge of SS, but also provides candidates for innovative diagnostics and therapeutics that could, with clinical development, improve the care of individuals with this illness.

## Figures and Tables

**Table 1:** Summary of non-coding RNAs reported in Sjögren’s syndrome patients.

Non-Coding RNA	Expression in SS Patients	Target/Association	Tissue/Cell	Comments	Ref.
miR-1248	Upregulated	IFN	Salivary gland	Overexpression affects both interferon and calcium signaling pathways	[17]
miR-513c-3p	Upregulated	XBP-1s and GRP78	LSG	Impact cellular proteostasis which regulate secretory function in LSG	[18]
miR-424–5p	Downregulated	ATF6α and SEL1L	LSG		[18]
miR-181a and miR-16	Downregulated	labial salivary pathological focus scores	LSG	Involved in SS pathogenesis through regulating La/SSB and Ro/SSA	[19]
miR-18b, miR-20a, miR-106a, miR-146b, miR-30, miR-17/92, miR-200, miR-let-7	Upregulated	Mucin O-glycosylation pathway	MSGBs	Cause dysfunction of salivary flow rates	[20]
miR-635, miR-372	Downregulated				
miR-181d-5p	Downregulated	TNF-α	LSG	Dysregulation could impact the glandular pro-inflammatory environment	[21]
miR-145-5p	Downregulated	Type 1 IFN, MUC1, TLR4	LSG	Has anti-inflammatory role	[22]
miR-3202	Downregulated	MMP2	PBMCs and Jurkat cell line	Has a protective function via inhibiting T cell infiltration from peripheral blood into the gland	[23]
miR-31-5p	Downregulated		PBMCs (CD4^+^)	Associated with energy metabolism	[24]
miR-146a-5p	Upregulated	ADAM17	PBMCs (Th17)	Plays a role in Th17 differentiation	[25]
miR-146a	Upregulated	CD80 TNF-α, IL-6, IL-1β MIP-1α, IP-10	PBMCs and Salivary gland	Elevates the phagocytic activity, suppresses the production of inflammatory cytokine, and could potentially modifies the regulation of T cells in autoimmune processes	[26,27]
miR-let-7d-3p	Downregulated	IL-17	PBMCs (CD4^+^)	Targeting the AKT1/mTOR signaling pathway, revealing a unique mechanism in pSS that may inform future treatment	[28]
miR-130a	Downregulated	MSK1	cDC2s	Inhibiting MSK1 can decrease the activity of cDC2s which consequently lowers the production of pro-inflammatory cytokines	[29]
mir-30b-5p	Downregulated	BAFF	PBMCs (B cells)	Correlated with decrease salivary flow in the gland	[30]
miR-744-5p	Upregulated	PELI3	PECs	Regulating eye inflammation in pSS	[31]
PVT1	Upregulated	Myc	PBMCs (CD19^+^, CD4^+^)	Engaged in altering the glycolytic metabolism and promoting cell growth	[32]
NEAT1	Upregulated	MAPK pathway	PBMCs (CD4^+^)	Elevated activation of the TCR pathway by PMA/ionomycin, suggested to be a contributing factor to the increased expression	[33]
LINC00426, TPTEP1-202	Downregulate	KATNAL1, HMGB1, UBE2L5, CCT8L2	PBMCs	Have a substantial correlation with markers of disease activity and regulate essential immunological processes	[34]
NRIR	Upregulated	RSAD2, CMPK2, RNF144A			
BISPR	Upregulated	PGLS, GTPBP3, MRPL34, PLVAP, CCDC194, NXNL1, DDA1, BST2, TMEM221			
circ-ZC3H6 and circ-IQGAP2	Upregulated	TFEC	MSGBs	Play important roles in pSS as non-invasive biomarkers associated with clinical characteristics	[35]
circ_0008301	Upregulated	TOLLIP	PBMCs	The expression level is high in patients associated with thrombocytopenia	[36]
lncRNAs	890 Upregulated 353 Downregulated	-	LSG	Demonstrating their potential as biomarkers and therapeutic targets for pSS, as well as the importance of their role in immunity response	[37]
mRNAs	1141 Upregulated 316 Downregulated				

Abbreviations: SS: Sjögren’s syndrome, Ref: References, PBMCs: peripheral blood mononuclear cells, cDC2s: circulating type-2 conventional dendritic cells, MSGBs: minor salivary gland biopsies, PECs: primary human conjunctival epithelial cells, LSG: labial salivary glands, circ: circular, NRIR: negative regulator of interferon response, BISPR: BST2 interferon stimulated positive regulator, ADAM17: A disintegrin and metalloprotease 17; NEAT1: nuclear enriched abundant transcript 1, Myc: myelocytomatosis oncogene, MSK1: Mitogen- and stress-activated protein kinase, BAFF: B-cell activating factor, KATNAL1: Katanin Catalytic Subunit A1 Like 1, HMGB1: High mobility group box 1, CCT8L2: Chaperonin Containing TCP1, Subunit 8 (Theta)-Like 2, RSAD2: Radical S-adenosyl methionine domain containing 2, CMPK2: Cytidine/Uridine Monophosphate Kinase 2, RNF144A: Ring Finger Protein 144A, PGLS: 6-Phosphogluconolactonase, GTPBP3: guanosine triphosphate binding protein 3, MRPL34: Mitochondrial ribosomal protein L34, PLVAP: Plasmalemma vesicle-associated protein, CCDC194: Coiled-coil domain containing 194, NXNL1: Nucleoredoxin Like 1, DDA1: DET1 And DDB1 Associated 1, TMEM221: Transmembrane protein 221, TFEC: Transcription Factor EC, TOLLIP: Toll interacting protein.
